# A Lower Gastrointestinal Bleeding Due to a Post-Traumatic Splenosis: “Wait and See” Represents a Feasible Attitude: A Case Report

**DOI:** 10.1097/MD.0000000000003555

**Published:** 2016-04-29

**Authors:** Fausto Famà, Giuseppa Giacobbe, Marcello Cintolo, Maria Gioffré-Florio, Socrate Pallio, Pierluigi Consolo

**Affiliations:** From the Department of Human Pathology, University Hospital of Messina, Messina, Italy.

## Abstract

Splenosis represents a benign condition due to an ectopic localization of splenic tissue caused by pathologic or traumatic spleen rupture. Generally, it is asymptomatic and incidentally diagnosed during imaging performed for other reasons. Occult gastrointestinal bleeding due to an extraperitoneal localization is a rare occurrence. Differential diagnosis may be very hard and includes benign and malignant neoplasms.

We describe the case of a 68-year-old Caucasian man that was admitted for an increasing lower gastrointestinal bleeding associated to a vague abdominal pain.

He was assessed by means of laboratory tests, as well as by endoscopic and radiological examinations, and successfully treated with an exclusive medical approach.

The patient was discharged on the ninth day and currently he is doing well.

This case shows that wait and see could prove a feasible attitude for the management of clinically stable patients.

## INTRODUCTION

Splenosis represents a benign condition characterized by an ectopic spreading of splenic tissue, brought about either by a pathologic or a traumatic spleen rupture. Splenosis is not frequently symptomatic and often is diagnosed only incidentally. The most common clinical events are idiopathic hemoperitoneum, bowel stenosis, and gastrointestinal (GI) bleeding, either spontaneous or traumatic.^1,2^ Differential diagnosis comes difficult for its radiological evidences apparently similar to metastatic lesions due to an occult neoplasia, benign or malignant tumors, and endometriosis.^3^ We report, to our knowledge for first time, a case of GI bleeding due to a splenosis-related small bowel involvement, treated by means of a conservative attitude.

## CASE REPORT

A 68-year-old Caucasian man arrived at the emergency unit of our hospital for an increasing lower GI bleeding that had begun few days before and also associated to a vague abdominal pain. Routine blood tests showed only a severe anemia with a hemoglobin (Hb) value of 6.4 g/dL, significantly decreased compared with the recent previous normal laboratory findings (Hb 16 g/dL); coagulation tests were normal. Physical examination did not reveal any abdominal abnormality. His medical history included an open splenectomy for the traumatic spleen rupture (motor vehicle accident) in 1991, a noninsulin-dependent diabetes mellitus, and an aspecific lymphocytosis with granulocytopenia, which was present even before the spleen trauma. Two years prior, a similar episode of lower GI bleeding with an Hb value of 9.1 g/dL occurred; nevertheless the patient chose to leave the hospital on the same day despite the physician's advice.

**FIGURE 1:**
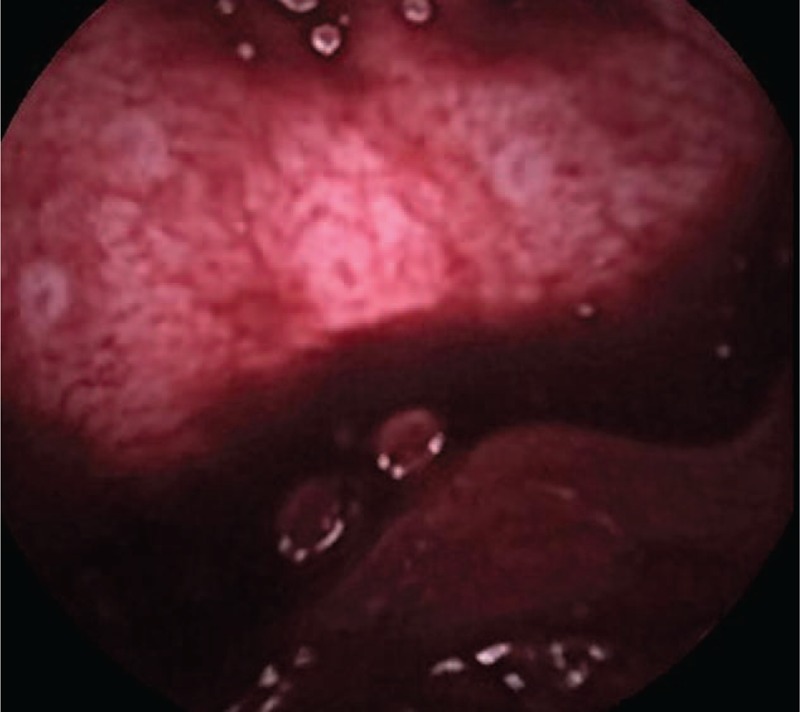
VCE on admission: abundant blood presence, particulary after crossed the jejunum, without evidence of the bleeding source. VCE = video capsule endoscopy.

The patient was admitted at the gastroenterology unit and about 4 hours later, after infusion of plasma expander and transfusion of 2 red blood cells units, his vital parameters improved and urgently was submitted to a whole colonoscopy that showed only noncomplicated diverticula with blood clots presence, without any active or visible bleeding source; moreover an esophagusgastroduodenoscopy (EGD) showed normal findings. Subsequently, to detect the bleeding occult site, a supplementary video capsule endoscopy (VCE) was carried out by means of PillCam SB2 (Given System). In the first video recording hour no blood traces were revealed; later they were more evident between the third and the fifth hour (Figure 1) and persistent until the shutdown of the examination. No evidence of mucosal lesions was present.

**FIGURE 2:**
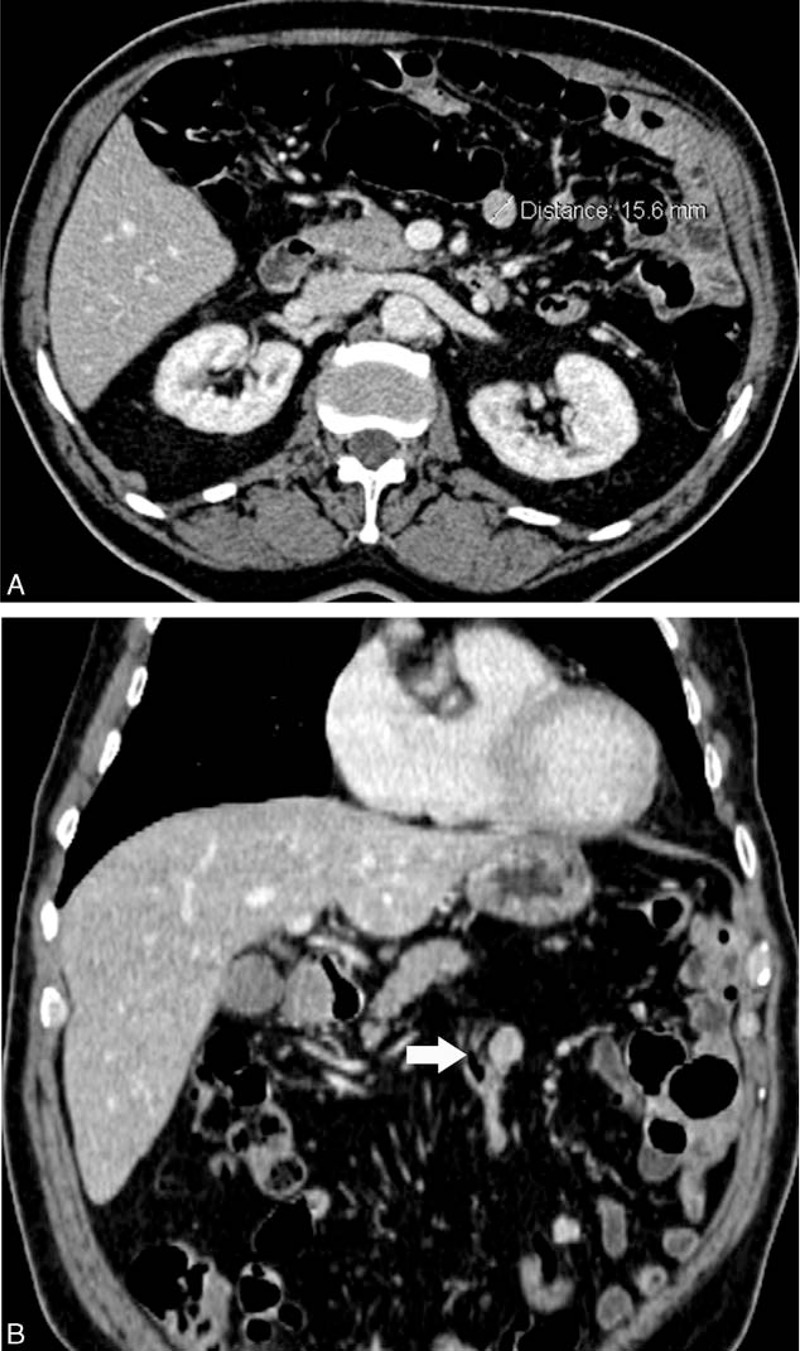
A, Contrast-enhanced CT, axial plane, showing a round mass (approximately 16 mm in its largest diameter) that presents a tight contact surface with a jejunal loop. B, Coronal plane: the white arrow is pointing out the splenosis mass. CT = computed tomography.

The ultrasound (US) scan detected a solid tissue in the splenic loggia, and an abdominal-enhanced computed tomography (CT) showed the presence of multiple intra- and extraperitoneal diffuse nodules, ranging between 1 and 3 cm, mainly localized on the left-side of the abdomen and pelvis (Figure 2A and B). Upon considering that one of these lesions, under 16 mm in its largest diameter, had a tight and large contact with a bundled and thick jejunum bowel loop, along with the endoscopic findings, we hypothesized the diagnosis of post-traumatic splenosis. A ^99m^Tc labeled heat-denatured red blood cell scintigraphy focused the attention on some limited areas with accumulation of the tracer in the left side of the abdomen, among these areas the biggest one was next to the splenic loggia, that, as matter of fact, supported clearly our presumed diagnosis.

**FIGURE 3:**
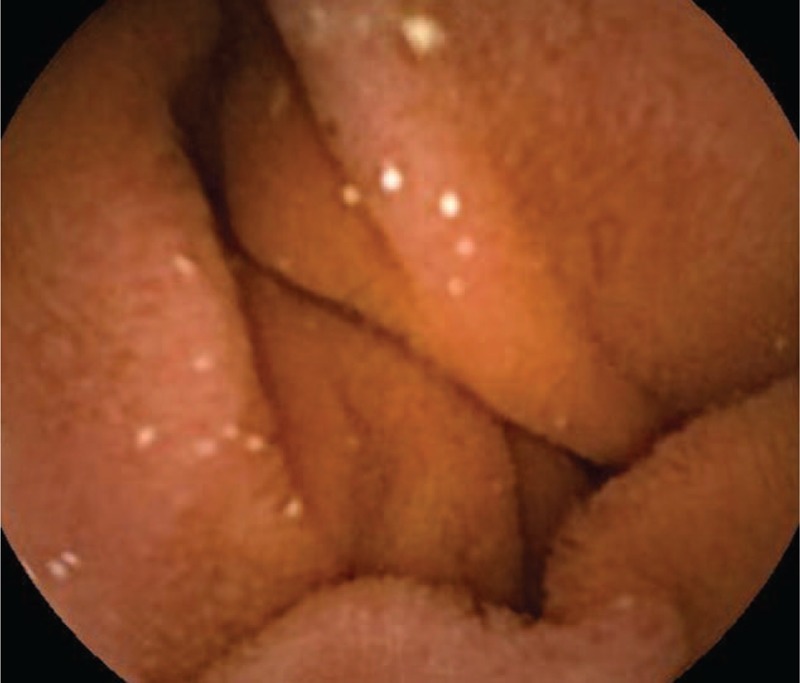
Follow-up VCE: normal finding. VCE = video capsule endoscopy.

The patient was transfused and gradually the GI bleeding reduced, without any need to carry out a surgical procedure thus rewarding our “wait and see” attitude. He was discharged on the 9th day, but was lost as outpatient in the follow-up. In the current year, the patient, fully asymptomatic, came back to our ward and was submitted to an endoscopic assessment, including a whole colonoscopy and a VCE exploration, both resulted completely negative (Figure 3); a supplementary enhanced CT scan showed an unchanged radiological pattern.

The above reported case is a rare occurrence of severe GI bleeding due to a post-traumatic splenosis nodule, that was tight to the wall of the small bowel, self-limited, and only medically treated.

## DISCUSSION

Splenosis is often an asymptomatic condition, diagnosed incidentally during US or radiological examination. GI bleeding either occult or not, spontaneous rupture of splenosis mass and abdominal pain are the most common occurrences in patients with intraperitoneal splenosis, whereas the extraperitoneal localization is rare.^4–6^ The heterotopic implantation foci are commonly multiple and mainly located in the greater omentum, in the peritoneal side of the small bowel and of the stomach. Sometimes the ectopic spleen tissue, not having a well-formed capsule differently from the normal spleen, grows in through the wall thickness up to the lumen of the GI tract, and may cause bleeding.^7^ US and radiological appearance pose issues of differential diagnosis with malignant and benign tumor masses, lymphomas, metastatic disease, and gastrointestinal stromal tumours,^5^ although its parenchymal tissue is totally comparable with the splenic imaging.^8,9^ Diagnosis is usually performed by means of US, CT scan, magnetic resonance imaging or, in other cases, according to their location, by endoscopy, useful even to exclude malignancies, ulcers, or any other common causes of bleeding;^10,11^ in this latter case the endoscopic appearance is often characterized by a raised mucosal area, which might be also hyperemic or ulcerated.^3,12^ In doubtful cases, a ^99m^Tc labeled heat-denatured red blood cell scintigraphy can be of use to perform diagnosis with a noninvasive examination, avoiding more aggressive exams.^13^ Surgery, laparoscopic or open, represents the treatment of choice, especially if symptomatic splenosis;^1,3,4^ When splenosis foci are difficult to be reached by surgery or for the patient's deteriorated general status, either with or without more relevant comorbidities, a transarterial embolization can be performed.^7^ When splenosis is asymptomatic, surgery is not indicated and a clinical follow-up is widely recommended by physicians.^14^

Although splenosis is not an uncommon occurrence in patients who have undergone splenectomy, literature does not report so many cases. Basile et al^3^ in 1989 described a case of acute massive GI bleeding due to foci of splenosis localized in the small bowel and treated by surgery. In the year 2000, Sikov et al^1^ reported a case of a male patient with chronic anemia and recurrent melena; endoscopic examinations and ^99m^Tc scintigraphy resulted negative. Diagnosis was made by abdominal angiography, which evidenced multiple foci of splenosis in the left colon and the small bowel. Multiple bowel resections with end-to-end anastomosis and a subtotal colectomy were performed.^1^ A similar case, also surgically treated, was marked by occult bleeding and complicated by intussusception.^15^

In 2010, Arroja et al^12^ published the first case of splenosis managed with a conservative treatment in a patient who complained melena and coffee grounds vomiting; the diagnosis was carried out by an EGD that showed a wide ulcer on the greater curvature of the stomach, later healed 3 weeks after his discharge.

Afterward, Obokhare et al^4^ reported a case of GI obstruction and bleeding due to a colonic localization of splenosis, successfully surgically treated. A Chinese group of authors in 2013, described another gastric localization of splenosis,^16^ and in the same year Alang^2^ published a very interesting case of acute bleeding due to a gastric splenosis; in that case the EGD showed a big clot adherent to the gastric fundus not removable by suctioning and irrigation. When blood appeared in the nasogastric tube placed after EGD, with worsening of vital parameters, a successful transarterial embolization to stop the bleeding was performed. Similar treatment was adopted by Leitz et al^7^ to treat an acute ileal bleeding splenosis-related.

In our case, the patient, although there was a surgical indication, was successfully medically treated and afterward we lost him at the follow-up. When we visited him again, 7 years later, the patient was in good clinical condition and had no more GI bleeding, which resolved independently; the imaging showed an unchanged condition compared with the previous CT scan. This case demonstrates, beyond the difficulty to manage these patients, that wait and see could prove a good strategy, both for asymptomatic and, in some selected cases, also in symptomatic patients; when surgery or embolization are not indicated or impracticable, provided that the patient be clinically stable and the bleeding reduced, that can be considered as a feasible attitude.
